# GRB2 Loss Attenuates Downstream TCR Effector Functions Despite Proximal ZAP-70 Hyper-Activation

**DOI:** 10.3390/biology15212027

**Published:** 2026-11-07

**Authors:** Inmaculada Vico-Barranco, Isaac Narbona-Sánchez, Luis M. Fernández-Aguilar, Ana García-García, Manuel Carrasco, Raquel Muñoz-Fernández, Mikel M. Arbulo-Echevarria, Enrique Aguado

**Affiliations:** 1 Department of Biomedicine, Biotechnology and Public Health, Universidad de Cádiz, 11002 Cádiz, Spain; inmaculada.vicobarranco@gmail.com (I.V.-B.); isaac.narbona@uca.es (I.N.-S.); luismi.fernandez@gm.uca.es (L.M.F.-A.); ana.garciagarcia2@alum.uca.es (A.G.-G.); manuel.carrasco@uca.es (M.C.); raquel.mfernandez@uca.es (R.M.-F.); miguel.martinezdearbuloechebarria@bio-gipuzkoa.eus (M.M.A.-E.); 2 Institute of Biomedical Research Cadiz (INIBICA), 11009 Cádiz, Spain

**Keywords:** GRB2, T-cell receptor signaling, THEMIS, CRISPR/Cas9 genome editing, signal transduction

## Abstract

The adapter protein GRB2 is central to T-cell receptor (TCR) signal transduction, yet its precise role in setting activation thresholds remains controversial. To clarify its functions, we generated stable *GRB2*-knockout (KO) Jurkat T-cell lines using CRISPR/Cas9 gene editing. Systematic analysis of TCR/CD3-stimulated *GRB2*-KO cells revealed widespread signaling hyper-activation: proximal ZAP-70 phosphorylation was drastically increased, and downstream signaling was markedly enhanced, evidenced by elevated ERK and PLC-γ1 phosphorylation along with heightened intracellular Ca^2+^ influx. In contrast, LAT phosphorylation was significantly decreased in GRB2-KO cells, indicating that GRB2 acts downstream or in parallel to constrain activation even when LAT phosphorylation is blunted. Intriguingly, loss of GRB2 was accompanied by reduced THEMIS protein expression, suggesting a potential link between GRB2 and THEMIS levels in human T cells. These findings support a role for GRB2 as an important negative gatekeeper of TCR signaling whose absence promotes hyperactivation despite reduced LAT phosphorylation, while to enhanced activation pathways while highlighting an intriguing association between GRB2 and THEMIS. Together, our results provide new mechanistic insights into the regulatory constraints governing human T-cell activation.

## Simple Summary

T cells are white blood cells that protect our bodies from infections and cancer by using surface sensors called T-cell receptors to detect threats. However, if T-cell activity is not carefully controlled, overactive responses can lead to autoimmune diseases, where immune cells attack healthy tissue. In this study, we investigated a specific helper protein called GRB2 to understand how T cells regulate their activity levels. Using precise gene-editing technology, we removed GRB2 from human T cells in the laboratory. Unexpectedly, deleting GRB2 caused the cells to become hyper-active, triggering stronger early activation signals and calcium movement inside the cell upon stimulation. Additionally, we found that cells lacking GRB2 also showed markedly reduced levels of another vital regulatory protein, THEMIS. These findings reveal that GRB2 acts as a crucial molecular brake to prevent T cells from overreacting. By showing how T-cell activation thresholds are maintained, this work provides valuable insights that could help researchers design better treatments for autoimmune disorders and improve immune system therapies.

## 1. Introduction

The cytosolic adapter growth factor receptor binding protein 2 (GRB2) is a ubiquitously expressed, evolutionarily conserved protein essential for cellular signaling. Structurally, GRB2 lacks enzymatic activity and consists of a central SH2 domain flanked by two SH3 domains [1,2]. The central SH2 domain binds to specific phosphorylated tyrosine residues, while the SH3 domains interact with proline-rich sequences of intracellular ligands, positioning GRB2 as a critical molecular bridge driving cell cycle progression, differentiation, and survival [3,4]. Consequently, dysregulation of GRB2 activity is implicated in various pathologies, including oncogenesis, cardiovascular diseases, and autoimmunity: its overexpression in multiple cancers accelerates oncogenic signaling and predicts poor prognosis [5], while in the cardiovascular system, it translates mechanical stress into MAPK activation, driving pressure-overload-induced hypertrophy and fibrosis [6]. Furthermore, GRB2 regulates CD4^+^ T-cell development and Th1/Th17 differentiation, directly modulating susceptibility to autoimmune neuroinflammation [7].

In T cells, GRB2 plays a pivotal role in regulating T-cell receptor (TCR) signaling. Upon TCR stimulation, the SH2 domain of GRB2 binds to phosphorylated tyrosine residues on the Linker for Activation of T cells (LAT) adapter [8], recruiting SH3-bound cytosolic ligands to the plasma membrane. Prominent among these ligands is the guanine nucleotide exchange factor Sos, which activates the Ras-MAPK pathway to promote cell proliferation and survival [9,10]. Because germline deletion of GRB2 is embryonically lethal [11], conditional knockout models have been utilized to study its specific role in T cells. These studies demonstrate that GRB2 is indispensable for both positive and negative thymocyte selection, acting as a quantitative factor that modifies the selection threshold [7,12,13].

While GRB2 is traditionally recognized as a positive transducer of activation signals, recent evidence highlights its role as a negative regulator of early TCR-triggered events. In vitro silencing of GRB2 in mature primary human CD4+ T cells and HuT78 T-cell lines resulted in the augmentation of proximal intracellular signals, including increased phosphorylation of Lck, ξ-chain, ZAP70, and SLP-76, alongside a paradoxical reduction in downstream effector functions such as PLC-γ1 phosphorylation, intracellular Ca^2+^ influx, and IL-2 and IFN-γ secretion [14]. Despite these findings, the precise molecular mechanisms by which GRB2 executes this dual regulatory function remain poorly understood.

A key candidate in this regulatory network is THEMIS (thymus expressed molecule involved in selection), a protein critical for setting the signaling thresholds that govern positive selection during thymic development [15,16]. Lacking intrinsic catalytic activity, THEMIS binds constitutively via its proline-rich region to the SH3 domain of GRB2, allowing its recruitment to the LAT signalosome upon TCR activation [13,17]. It has been proposed that GRB2 acts as a structural bridge to form a THEMIS-GRB2-SHP1 trimolecular complex, thereby modulating the activity and localization of the SHP1 phosphatase [18,19]. However, the exact nature of this feedback mechanism remains highly controversial: some models support positive feedback via SHP1 inhibition [20,21], while others point to a negative feedback loop via SHP1 activation [22].

Resolving the precise cooperative mechanisms involving GRB2 and THEMIS within the TCR signaling cascade is critical to understanding how adaptive immune responses are finely tuned. Recent efforts, including work by Ruminsky et al., have established GRB2-deficient human Jurkat T-cell derivatives using gene editing to explore adapter networks in human T cells [23]. Building upon this genetic foundation, a systematic mapping of early, proximal signaling kinetics, threshold modulation, and adapter stability in complete GRB2-null human T-cell lines is required to clarify how GRB2 dictates signal output. To this end, the primary objective of this study was to systematically delineate how the complete loss of GRB2 impacts early TCR-mediated signal propagation. To address this objective, we generated stable *GRB2*-knockout Jurkat T-cell lines using CRISPR/Cas9 genomic editing to conduct a high-resolution analysis of TCR/CD3-triggered cascade events. We demonstrate that total loss of GRB2 leads to a hyper-activation of T-cell signaling, characterized by a drastic increase in ZAP-70, ERK, and PLC-*γ*1 phosphorylation alongside elevated intracellular Ca^2+^ influx upon CD3 stimulation, even though LAT phosphorylation is reduced. Intriguingly, we observed a downregulation of THEMIS protein expression in GRB2-deficient cells. Together, these findings redefine GRB2 as a critical negative gatekeeper of T-cell activation whose absence uncouples LAT phosphorylation from downstream hyper-activation, while uncovering a novel dependency between GRB2 and THEMIS stability in human T cells.

## 2. Materials and Methods

### 2.1. Cell Culture, Cloning and Lentiviral Transfection

The human T-lymphocyte Jurkat cell line (clone E6-1) was obtained from European Collection of Authenticated Cell Cultures (ECACC) and routinely tested for mycoplasma contamination. Jurkat T cells were maintained in complete RPMI 1640 medium (Hyclone, Logan, UT, USA) supplemented with 10% fetal calf serum (FCS) (Lonza, Basel, Switzerland), Penicillin-Streptomycin (100 U/mL, Biowest, Nuaillé, France), and 2 mM L-glutamine (Biowest) at 37 °C in a humidified atmosphere with 10% CO_2_.

### 2.2. Targeted Deletion of the GRB2 Gene in Jurkat Cells Using CRISPR/Cas9 Nickase

To minimize off-target editing, *GRB2*-deficient Jurkat cell lines were generated using a paired CRISPR/Cas9 nickase (Cas9n, D10A mutant) approach. We utilized the Cas9n variant because requiring two adjacent single-strand nicks to generate a double-strand break significantly reduces off-target cleavage compared to wild-type Cas9 [24]. Single-guide RNAs (sgRNAs) targeting exon 2 of GRB2 (which contains the primary translation initiation codon) were selected and evaluated for off-target effects using the CHOPCHOP bioinformatic tool (https://chopchop.cbu.uib.no/) [25]: sgRNA1: 5′-CGACGAGCTGAGCTTCAAAA-3′ and sgRNA2: 5′-TTTGAAGTCATATTTGGCGA-3′. Complementary oligonucleotides containing BbsI-compatible overhangs (Forward: 5′-CACCG…-3′; Reverse: 5′-AAAC…C-3′) were annealed and cloned into the BbsI sites of the pSpCas9n(BB)-2A-Puro vector (PX462 V2.0; Addgene plasmid #62987). The resulting constructs were co-transfected into Jurkat cells via electroporation.

Parental Jurkat cells were co-electroporated with 1 μg of each construct using the AMAXA Cell Line Nucleofector Kit V (Lonza, Basel, Switzerland) according to the manufacturer’s protocol. Cells were cultured for 72 h post-electroporation to allow transient Cas9n expression, targeted double-nick cleavage, and subsequent genomic locus repair. Genomic editing efficiency was evaluated by T7 Endonuclease I (T7E1) cleavage assay (New England Biolabs, Ipswich, MA, USA) on PCR-amplified *GRB2* exon 2 sequences. Polyclonal pools exhibiting targeted cleavage were subjected to limiting dilution in 96-well plates to isolate single-cell clones. Monoclonal lines were initially screened for loss of GRB2 protein expression via Western blot analysis.

To characterize the precise allelic mutations in selected GRB2-deficient clones, target loci were analyzed at both the genomic DNA (gDNA) and cDNA levels. Target regions were amplified using Taq DNA polymerase (Thermo Fisher Scientific, Waltham, MA, USA) with specific primers for gDNA (MM-Fw: 5′-AGTGGACCGTATTTGGTGCTGG-3′; MM-Rv: 5′-CACCACACATCACACCAACTCACTT-3′) and cDNA (RT-Fw: 5′-GTGGCATTGTGTGTCCCAGAGT-3′; RT-Rv: 5′-CAGAGGCAGCTTGTGGGTTTAA-3′). Purified amplicons were TA-cloned into the pCR2.1 vector (Invitrogen, Carlsbad, CA, USA) and transformed into E. coli Stbl3 cells. DNA purified from multiple bacterial colonies was sequenced by Sanger sequencing to ensure complete coverage of all mutated alleles.

### 2.3. Generation of Reconstitution Constructs and Lentiviral Transduction

To generate GRB2 reconstitution construct, full-length *GRB2* cDNA was cloned upstream of an IRES cassette into a pENTR11 entry vector (Invitrogen), as previously described [26]. Coding sequence integrity was verified by Sanger sequencing. The expression cassette was subsequently recombined into the self-inactivating (SIN) lentiviral vector pHR’SINcPPT-Hygro-mCherry using Gateway LR Clonase II Enzyme Mix (Invitrogen).

Lentiviral particles were produced in HEK293T cells by co-transfection of the transfer vector alongside the envelope plasmid (PMD2.G) and packaging plasmid (p8.91). Lentiviral supernatants were harvested and used to transduce *GRB2*-deficient Jurkat cells to restore GRB2 expression. To ensure robust proviral genome integration and transgene expression prior to selection, antibiotic selection with hygromycin (20 μg/mL) was initiated 72 h post-transduction. Expression of mCherry was used as a reporter of transduction efficiency using flow cytometry (CytoFLEX, Beckman Coulter, Brea, CA, USA). In all experiments, the proportion of mCherry-positive cells exceeded 80%.

### 2.4. Preparation of Cell Lysates and Western Blotting

Lentivirally transduced J.GRB2 cells were starved in serum-free RPMI 1640 for 18 h prior to stimulation to eliminate baseline background phosphorylation induced by serum factors and synchronize cells into a resting signaling state. Cells were subsequently stimulated with anti-CD3 monoclonal antibody (mAb, clone OKT3; 1 μg/mL; eBioscience, San Diego, CA, USA) at 37 °C, for the indicated time points (0, 3, 10 and 30 min) to capture both peak early proximal signaling kinetics (3 min) and subsequent signal propagation/attenuation dynamics (10–30 min). Cells were then lysed at 2.0 × 10^7^ cells/mL in 2× Laemmli buffer, followed by incubation at 99 °C for 5 min and sonication.

For Western blotting, whole-cell lysates were separated by SDS-PAGE and transferred onto PVDF membranes. Membranes were blocked and incubated with the indicated primary antibodies, followed by incubation with the appropriate IRDye 800CW-conjugated secondary antibodies (Li-Cor, Lincoln, NE, USA). Reactive protein bands were visualized using the Odyssey CLx Infrared Imaging System (Li-Cor, Lincoln, NE, USA). For reprobing, PVDF membranes were incubated with WB Stripping Solution (Nacalai Tesque, Kyoto, Japan) for 10 min at room temperature, followed by washing in Tris-buffered saline with Tween-20 (TTBS).

### 2.5. Ca^2+^ Mobilization Assay

Measurement of intracellular free Ca^2+^ was carried out using Indo-1 AM (acetoxymethyl ester, 2 μM; Molecular Probes, Invitrogen) as previously described [26]. Calcium kinetics were measured using a Synergy MX Multi-Mode Reader (Biotek Instruments, Winooski, VT, USA) at 37 °C. Cells were excited at a wavelength of 340 nm, and emission fluorescence at 405 and 485 nm was recorded alternately at 4 s intervals. Intracellular Ca^2+^ mobilization was determined as the ratio of emission intensities (F_405_/F_485_).

### 2.6. Flow Cytometry and Surface Phenotyping

Surface expression of CD3, CD6 and CD69 was quantified by flow cytometry. Cells (0.5 × 10^6^) were washed twice with ice-cold staining buffer (PBS containing 1% BSA) and incubated with fluorophore-conjugated primary antibodies: APC anti-human CD3 (clone OKT3), PE anti-human CD6 (clone BL-CD6) and Alexa Fluor 700 anti-human CD69 (clone FN50) (all from BioLegend San Diego, CA, USA) for 30 min at 4 °C in the dark. For CD69 upregulation assays, cells were left unstimulated or stimulated with anti-CD3 mAb (OKT3; 1 μg/mL) for 18 h prior to staining, an optimal kinetic timeframe established for de novo transcriptional activation and stable cell surface accumulation of CD69 without inducing cell mortality. Following incubation, cells were washed twice with staining buffer and acquired on a CytoFLEX flow cytometer (Beckman Coulter). Flow cytometry data were analyzed using FlowJo software version 10.8.1 (BD Biosciences, Franklin Lakes, NJ, USA).

### 2.7. Real-Time Quantitative RT-PCR (RT-qPCR)

Total RNA was extracted from cells using the TRIzol™ LS Reagent (Invitrogen) according to the manufacturer’s protocol. RNA was reverse transcribed using qScript™ cDNA Synthesis kit (QuantaBio, Beverly, MA, USA) and used for conventional and quantitative PCR. Quantitative RT-PCR (RT-qPCR) assays were performed in technical triplicates using CFX Connect Real-Time PCR Detection System (Bio-Rad, Hercules, CA, USA). Amplifications were carried out using PerFeCTa SYBR Green FastMix (QuantaBio, Beverly, MA, USA). Thermal cycling conditions for *THEMIS* mRNA were initial denaturation at 95 °C for 30 s, followed by 40 cycles of 95 °C for 5 s, 54 °C for 15 s, and 72 °C for 10 s. To verify specific amplification and rule out primer-dimer formation, melting curve analysis was conducted from 65 °C to 95 °C with 0.5 °C increments. Relative expression levels of *THEMIS* were normalized to *HPRT1*. Specific primers used were as follows: *THEMIS* (Forward: 5′-CATCTATCTTGAAGGCTC-3′; Reverse: 5′-TGAGACCAGTAATTTTAATC-3′), *HPRT1* (Forward: 5′-GGCGTCGTGATTAGTGATGATG-3′; Reverse: 5′-GAATTTATAGCCCCCCTTGAGC-3′).

### 2.8. Statistical Analysis

All data are expressed as mean ± standard error of the mean (SEM) from at least three independent biological experiments, with technical replicates specified in the corresponding figure legends. Statistical evaluations were performed using GraphPad Prism software version 10.5.0 (GraphPad Software, San Diego, CA, USA).

Differences between wild-type and *GRB2*-deficient groups across time courses were analyzed using two-way analysis of variance (ANOVA) followed by Sidak’s or Tukey’s multiple comparisons test. Comparisons between two groups were evaluated using the two-tailed unpaired Student’s *t*-test or Mann–Whitney U test as appropriate. Statistical significance was defined as * *p* < 0.05, ** *p* < 0.01, *** *p* < 0.001, **** *p* < 0.0001.

## 3. Results

### 3.1. GRB2 Gene Deletion

**Figure 1:**
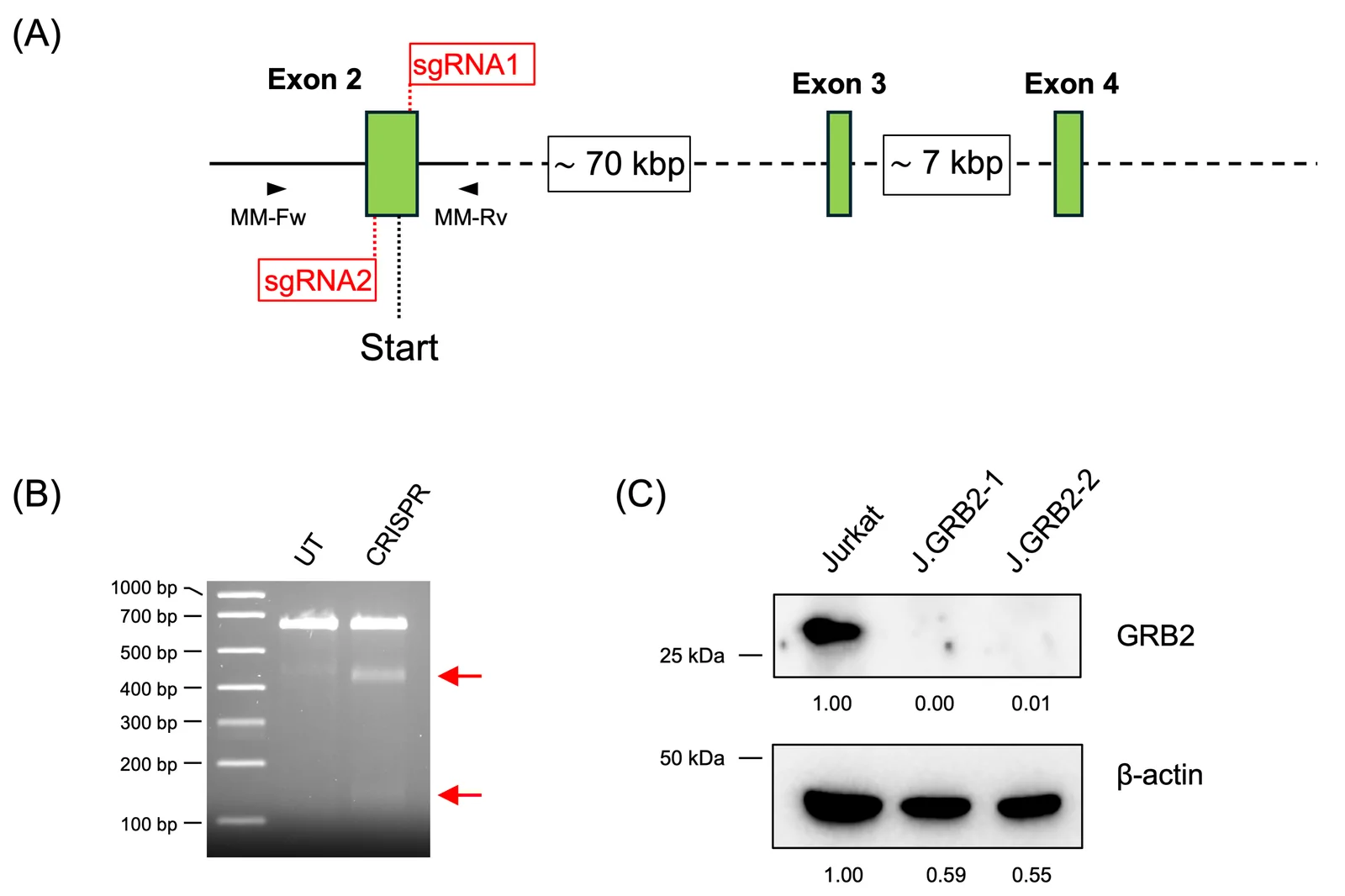
**CRISPR/Cas9n editing strategy for the knockout of the *****GRB2***** gene in Jurkat cells. **(**A**) Schematic of the *GRB2* genomic locus showing exons 2, 3, and 4 (green boxes). Target sites for the two single-guide RNAs (sgRNA1 and sgRNNA2) within exon 2 and the binding locations of PCR primers MM-Fw and MM-Rv are indicated. (**B**) T7 endonuclease I (T7E1) mismatch detection assay. Genomic DNA isolated from untreated parental Jurkat (UT) and electroporated Jurkat cells (CRISPR) was PCR-amplified with primers MM-Fw and MM-Rv and subjected to T7E1 digestion. Red arrows indicate cleavage products resulting from T7E1 recognition of mismatch loops, confirming NHEJ-mediated indels at the target locus. (**C**) Western blot analysis of GRB2 expression in edited Jurkat clones. Parental Jurkat cells (JKT) and two selected GRB2-deficient single-cell clones (J.GRB2-1 and J.GRB2-2) were lysed and analyzed by immunoblotting using the indicated GRB2-specific antibodies (upper panel). Membranes were stripped and reprobed with anti-β-actin antibodies to serve as a loading control (bottom panel). Numerical values below the blots represent relative band intensity values for each lane. The full Western blot figures can be found in the Supplementary Materials.

To disrupt the GRB2 gene in Jurkat cells while minimizing off-target editing, we utilized a paired CRISPR/Cas9 nickase (Cas9n) strategy targeting exon 2, which contains the primary translation initiation codon (Figure 1A). T7 endonuclease I (T7E1) cleavage assays on PCR-amplified genomic DNA confirmed targeted gene editing within the transfected cell pool (Figure 1B). Subsequent Western blot screening of single-cell clones isolated by limiting dilution identified two independent monoclonal lines (designated J.GRB2-1 and J.GRB2-2) exhibiting a complete loss of GRB2 protein expression (Figure 1C).

**Figure 2:**
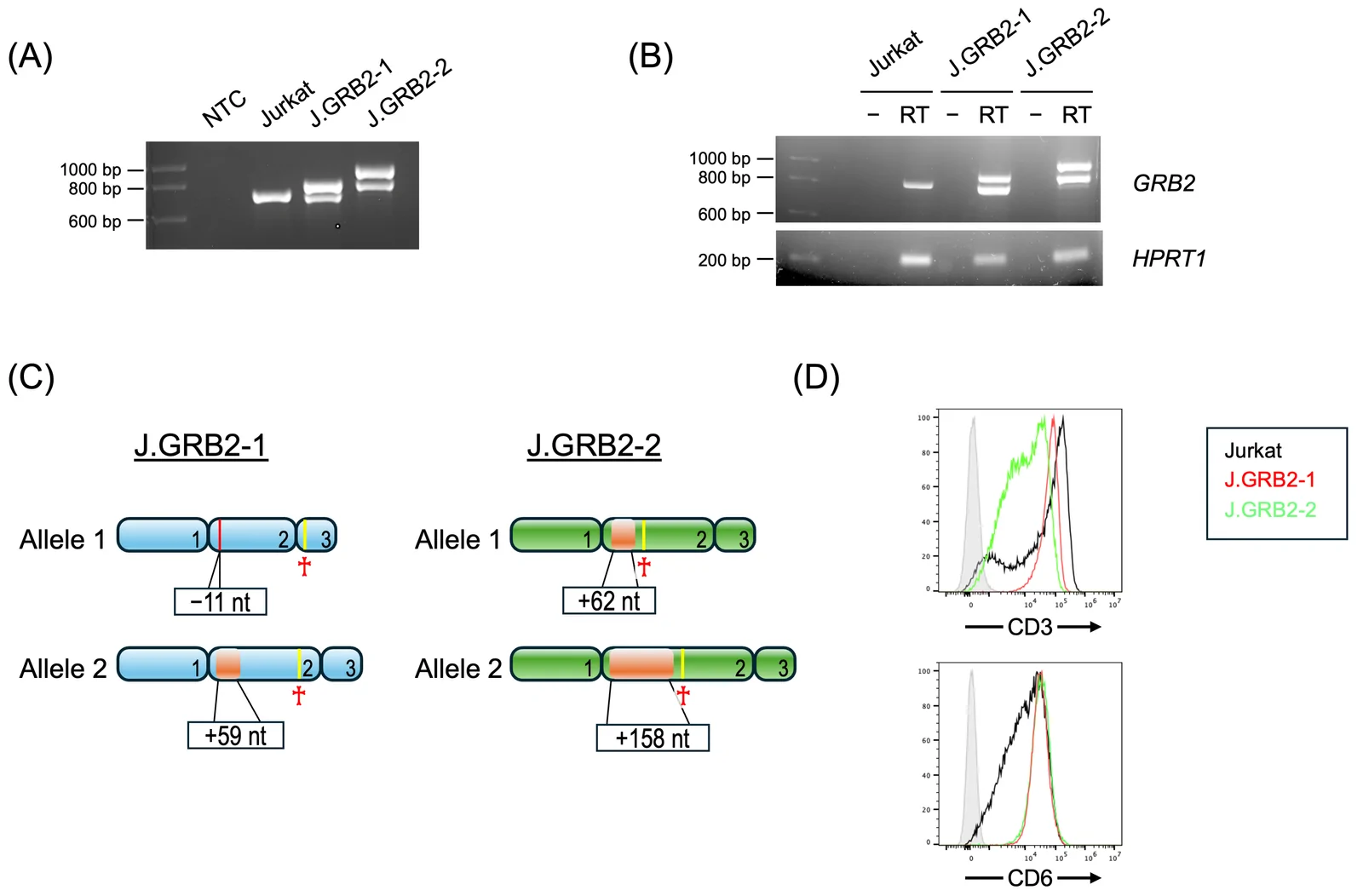
**Molecular characterization of CRISPR-Cas9-mediated *****GRB2***** gene disruption at the genomic DNA and mRNA levels.** (**A**) PCR analysis of the *GRB2* genomic locus. Genomic DNA isolated from parental Jurkat (JKT), J.GRB2-1, and J.GRB2-2 cell lines was amplified using the primer pair MM-Fw/MM-Rv. A no-template control (NTC) was included as a negative control. Band sizes correspond to the respective wild-type and edited alleles of the GRB2 gene. (**B**) RT-PCR analysis of *GRB2* transcript expression. Total RNA from Jurkat, J.GRB2-1, and J.GRB2-2 cells was analyzed by RT-PCR using GRB2-specific primers (RT-Fw/RT-Rv) and HPRT1 primers as an internal loading control. Control reactions without reverse transcriptase (“−”) confirm the absence of genomic DNA contamination. (**C**) Schematic representation of CRISPR-induced indels in the *GRB2* gene. The structure of the first three exons is shown for both alleles of J.GRB2-1 and J.GRB2-2 cell lines. Insertions (+) or deletions (–) in nucleotides (nt) are indicated for each allele, including an 11-nt deletion spanning nucleotides +42 to +52 (relative to the ATG start codon, where +1 corresponds to ‘A’) in clone J.GRB2-1. Red crosses (♰) designate the location of the first premature stop codon introduced by frameshift mutations. (**D**) Flow cytometric analysis of CD3 (top) and CD6 (bottom) surface expression in Jurkat (black line), J.GRB2-1 (red line) and J.GRB2-2 (green line) cells. The grey filled histogram represents the isotype control. One representative experiment is shown.

To characterize the precise genetic alterations in these two knockout lines, we amplified the exon 2 region of the GRB2 gene from genomic DNA using specific primers. PCR analysis revealed distinct band profiles for clones J.GRB2-1 and J.GRB2-2 compared to wild-type Jurkat cells, indicating that unique indels had occurred in the alleles of each clone (Figure 2A). Specifically, clone J.GRB2-1 yielded one band of wild-type size and one larger band, suggesting heterozygous modifications. In contrast, clone J.GRB2-2 displayed two distinct, larger bands, indicating different insertional modifications in both alleles. To determine whether these genomic modifications permitted transcription and whether the resulting mRNA transcripts reflected these size differences, we performed RT-PCR on total RNA extracted from wild-type and clone cells. Consistent with our genomic DNA findings, the amplified GRB2 cDNAs from both clones exhibited size variations that mirrored their respective genomic alterations (Figure 2B).

To determine the precise sequence of the indels responsible for these size variations, we cloned the genomic DNA and cDNA amplicons from each line into the pCR™2.1 vector. Multiple clones were sequenced to ensure complete coverage of both alleles for each cell line. Sequencing revealed distinct, frame-shifting alterations in all target alleles (Figure 2C). Specifically, clone J.GRB2-1 harbored an 11-nt deletion spanning nucleotides +42 to +52 (relative to the ATG start codon, where +1 corresponds to the ‘A’) on one allele (introducing a premature stop codon at the start of exon 3) and a 59-nt insertion on the other (resulting in a premature stop codon at the end of exon 2). In clone J.GRB2-2, we identified a 62-nt insertion on one allele and a 158-nt insertion on the other, both of which introduced premature stop codons within exon 2. Ultimately, these genomic disruptions successfully aborted translation, leading to the complete loss of GRB2 protein expression in both knockout lines.

Because CD3 and CD6 are critical regulators of the intensity and duration of T-cell receptor (TCR) signaling [27], we next evaluated their surface expression on both knockout clones via flow cytometry. Flow cytometric analysis revealed that J.GRB2-1 cells featured a higher percentage of CD3^+^ cells compared to parental Jurkat cells, albeit with a lower overall expression intensity (Figure 2D, upper panel). In contrast, the J.GRB2-2 clone exhibited reductions in both the percentage of CD3^+^ cells and their signal intensity (Figure 2D, upper panel). Regarding CD6 expression, we observed negligible differences in the percentage of positive cells among wild-type Jurkat, J.GRB2-1, and J.GRB2-2 lines. However, the surface expression intensity of CD6 was notably elevated in both GRB2-deficient clones relative to parental cells (Figure 2D, lower panel). Based on these surface marker profiles (particularly the more favorable CD3 expression pattern in J.GRB2-1) we selected the J.GRB2-1 clone (hereafter referred to as J.GRB2) as our representative model for subsequent downstream TCR signaling assays. Collectively, these genomic and phenotypic characterizations validate the successful establishment of a robust, well-defined GRB2-null cellular model.

### 3.2. Impact of GRB2 Deletion on Early TCR Signaling

**Figure 3:**
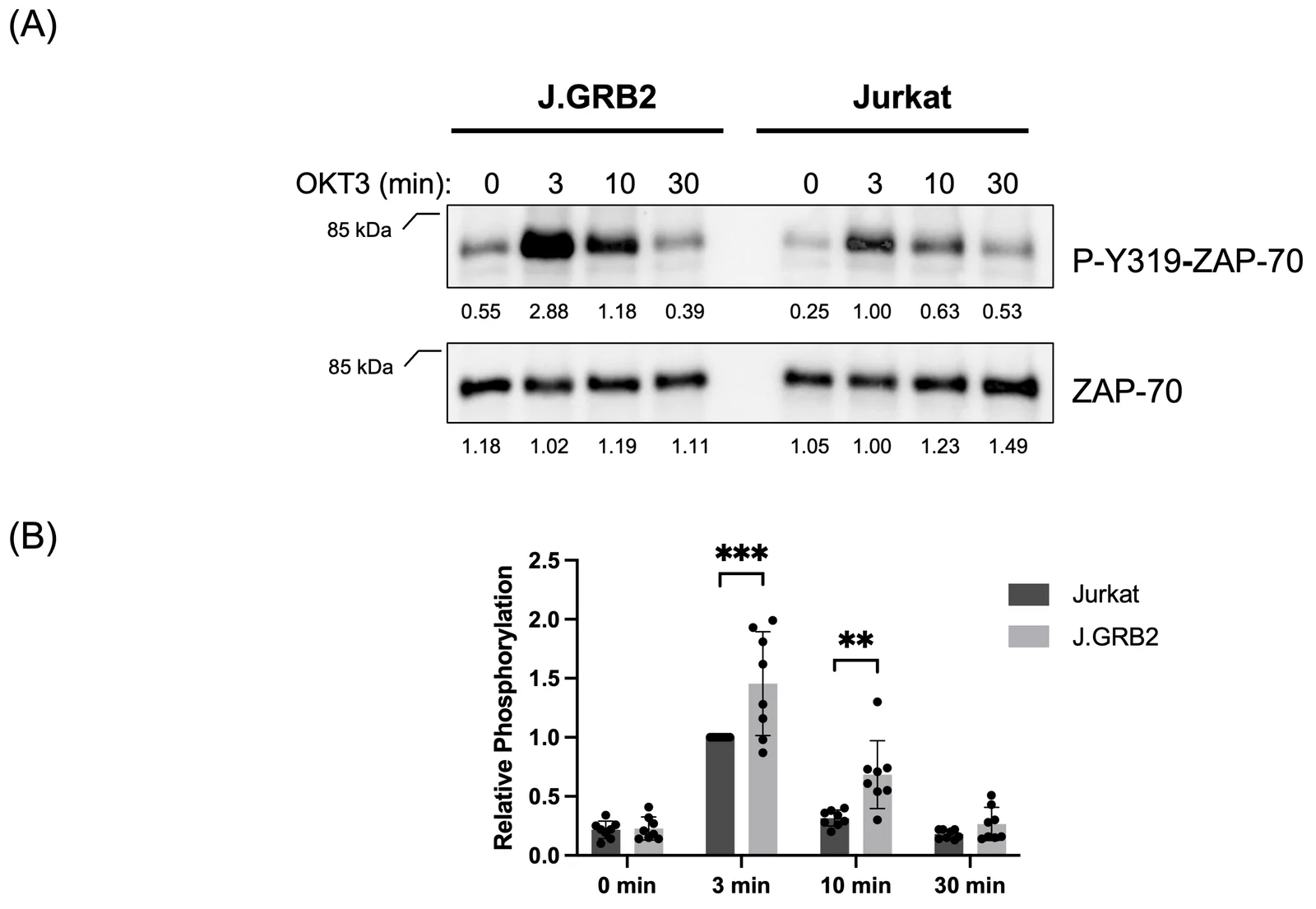
**GRB2 deficiency enhances ZAP-70 Tyr319 phosphorylation upon TCR engagement.** Parental Jurkat and GRB2-deficient (J.GRB2) cells were stimulated with anti-CD3 monoclonal antibody (OKT3) for the indicated time points (0, 3, 10, and 30 min). (**A**) Representative Western blot analysis of whole-cell lysates assessing ZAP-70 phosphorylation at Tyr319 (P-Y319 ZAP70) and total ZAP-70 expression (ZAP-70) as a loading control. Numerical values below the blots represent relative band intensity values for each lane. (**B**) Densitometric quantification of ZAP-70 Tyr319 phosphorylation normalized to total ZAP-70 levels across the stimulation time course. Data are presented as mean ± SEM of eight independent experiments (*n* = 8). Statistical significance between parental Jurkat and J.GRB2 cells at each time point was determined by two-way ANOVA (** *p* < 0.01; *** *p* < 0.001).

Following T-cell receptor (TCR) engagement, the protein tyrosine kinase ZAP-70 undergoes rapid phosphorylation on key regulatory residues. In particular, phosphorylation of tyrosine 319 (Tyr319), located in interdomain B, is critical for relieving ZAP-70 autoinhibition and propagating downstream signaling from the TCR/CD3 complex [28]. To determine whether GRB2 deficiency impacts early proximal TCR signaling or alters ZAP-70 activation kinetics, we evaluated the phosphorylation status of ZAP-70 Tyr319. To test this, parental Jurkat cells and J.GRB2 cells were stimulated at 37 °C with anti-CD3 monoclonal antibody, and whole-cell lysates were obtained at 0, 3, 10, and 30 min post-stimulation. Anti-CD3 stimulation induced a rapid increase in ZAP-70 Tyr319 phosphorylation in both parental Jurkat and J.GRB2 cells (Figure 3A). However, the magnitude of ZAP-70 Tyr319 phosphorylation was markedly enhanced in J.GRB2 cells relative to control cells across the stimulation time course. Densitometric quantification across eight independent experiments confirmed a statistically significant elevation in ZAP-70 activation in GRB2-deficient cells (Figure 3B). Together, these results demonstrate that GRB2 deficiency hyper-activates TCR-proximal signaling, highlighting an important role for GRB2 in exerting negative feedback control on early ZAP-70 activation.

**Figure 4:**
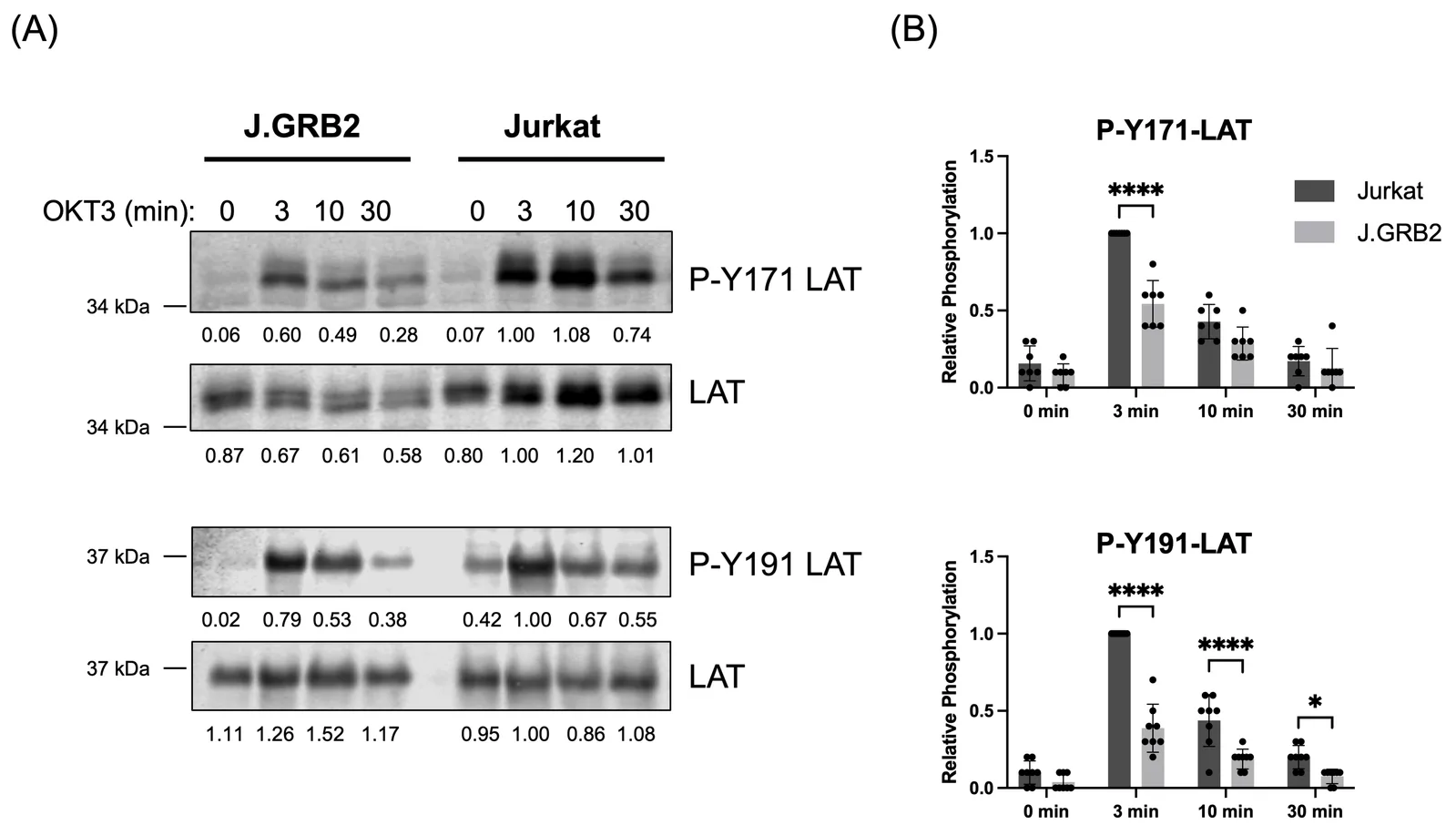
**GRB2 deficiency impairs phosphorylation of both LAT Tyr171 and Tyr191 following TCR engagement.** Parental Jurkat and GRB2-deficient (J.GRB2) cells were stimulated with anti-CD3 monoclonal antibody (OKT3) for the indicated time points (0, 3, 10, and 30 min). (**A**) Representative Western blot analysis of whole-cell lysates assessing LAT phosphorylation at Tyr171 (P-Y171 LAT) and Tyr191 (P-Y191 LAT), with total LAT expression (LAT) shown as a loading control. Numerical values below the blots represent relative band intensity values for each lane. (**B**) Densitometric quantification of LAT Tyr171 (top) and Tyr191 (bottom) phosphorylation normalized to total LAT levels across the stimulation time course. Data are presented as mean ± SEM of seven independent experiments (*n* = 7). Statistical significance between parental Jurkat and J.GRB2 cells at each time point was determined by two-way ANOVA (* *p* < 0.05; **** *p* < 0.0001).

**Figure 5:**
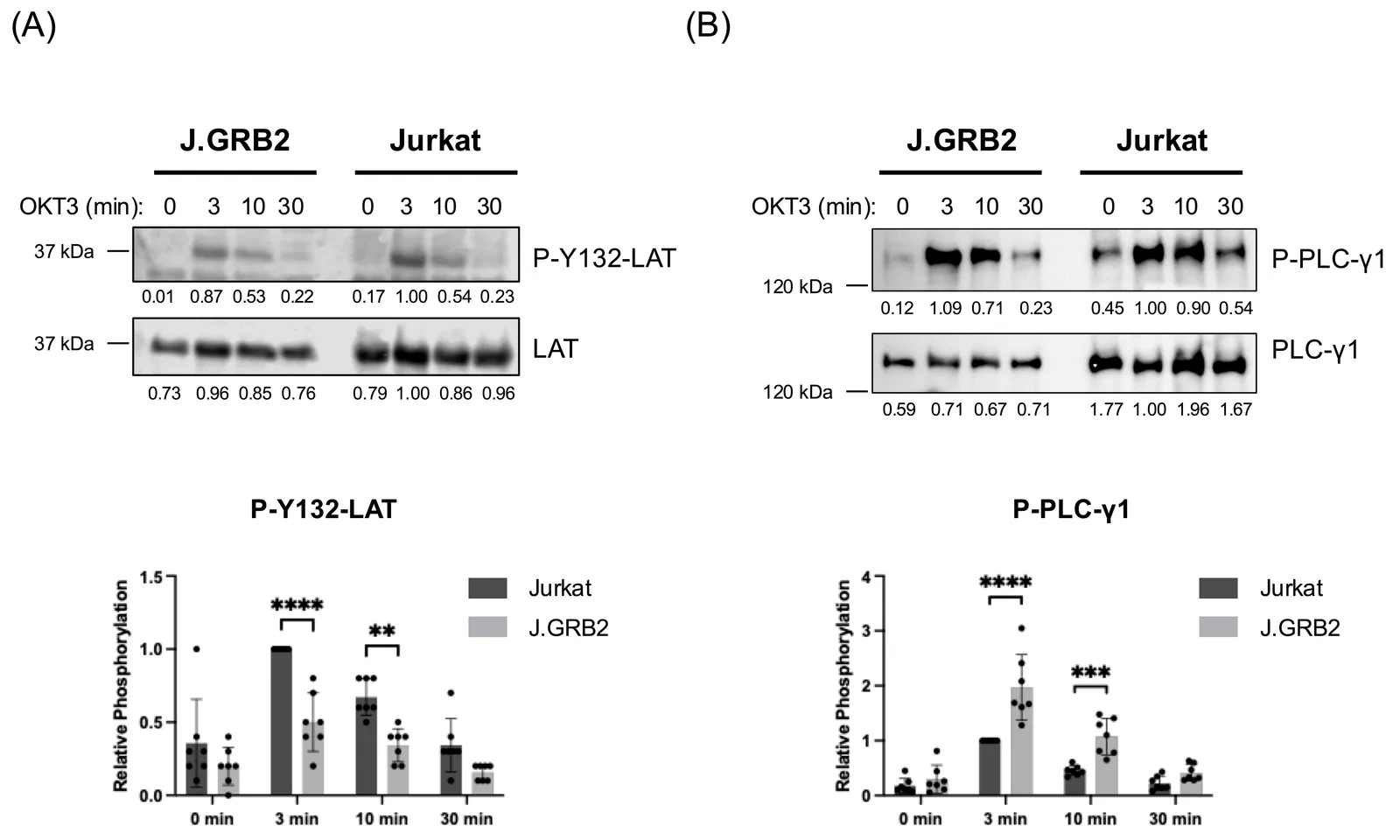
**Loss of GRB2 impairs LAT Tyr132 phosphorylation despite enhanced PLC-γ1 activation.** Parental Jurkat and GRB2-deficient (J.GRB2) cells were stimulated with OKT3 monoclonal antibody across a 30-min time course. (**A**) Representative immunoblots (top) and corresponding densitometric analysis (bottom, *n* = 6) of LAT Tyr132 phosphorylation (P-Y132-LAT) relative to total LAT protein. (**B**) Representative immunoblots (top) and corresponding densitometric analysis (bottom, *n* = 11) of PLC-γ1 phosphorylation (P-PLC-γ1) relative to total PLC-γ1 protein. Sub-band numerical values indicate relative lane signal intensities. Bar graphs express mean ±SEM of seven independent experiments. Group differences across time points were analyzed by two-way ANOVA (** *p* < 0.01; *** *p* < 0.001; **** *p* < 0.0001).

We next examined whether GRB2 deletion affects downstream propagation of TCR signals at the level of the LAT signalosome. Densitometric quantification of LAT phosphorylation at tyrosine 171 (P-Y171 LAT) revealed a significant reduction in GRB2-deficient (J.GRB2) cells compared to control Jurkat cells following CD3 stimulation (Figure 4A), reaching statistical significance at 3 min post-stimulation (**** *p* < 0.0001; Figure 4B). A similar marked decrease was observed for LAT phosphorylation at tyrosine 191 (P-Y191 LAT) across time points (*** *p* < 0.0001 at 3 and 10 min, *p* < 0.05 at 30 min; Figure 4B). We also specifically evaluated the phosphorylation status of LAT Tyr132, a key residue required for the efficient recruitment and subsequent activation of phospholipase C-γ1 [29,30,31]. Consistent with the reductions observed at Tyr171 and Tyr191, removal of the GRB2 adaptor significantly impaired LAT Tyr132 phosphorylation (Figure 5A). Quantitative analysis confirmed a significant reduction in P-Tyr132 levels in J.GRB2 cells at 3 min (**** *p* < 0.0001) and 10 min (*p* < 0.01) post-stimulation (Figure 5A, bottom panel). Strikingly, despite this widespread reduction across all major LAT phosphorylation sites (Y132, Y171, and Y191), analysis of PLC-γ1 activation demonstrated that GRB2 deficiency significantly enhanced PLC-γ1 phosphorylation upon CD3 stimulation (Figure 5B). Quantitative analysis confirmed a robust increase in P-PLC-γ1 levels in J.GRB2 cells, reaching statistical significance at the peak 3-min (**** *p* < 0.0001) and 10-min (*** *p* < 0.001) time points (Figure 5B, bottom panel). Together, these findings indicate that while GRB2 is required for optimal phosphorylation across the entire LAT signalosome, its absence uncouples diminished LAT phosphorylation from downstream PLC-γ1 hyperactivation, revealing a key repressive role for GRB2 in setting the activation threshold of downstream signaling cascades.

### 3.3. GRB2 Deficiency Enhances Anti-CD3-Induced Ca^2+^ Influx and ERK Phosphorylation

**Figure 6:**
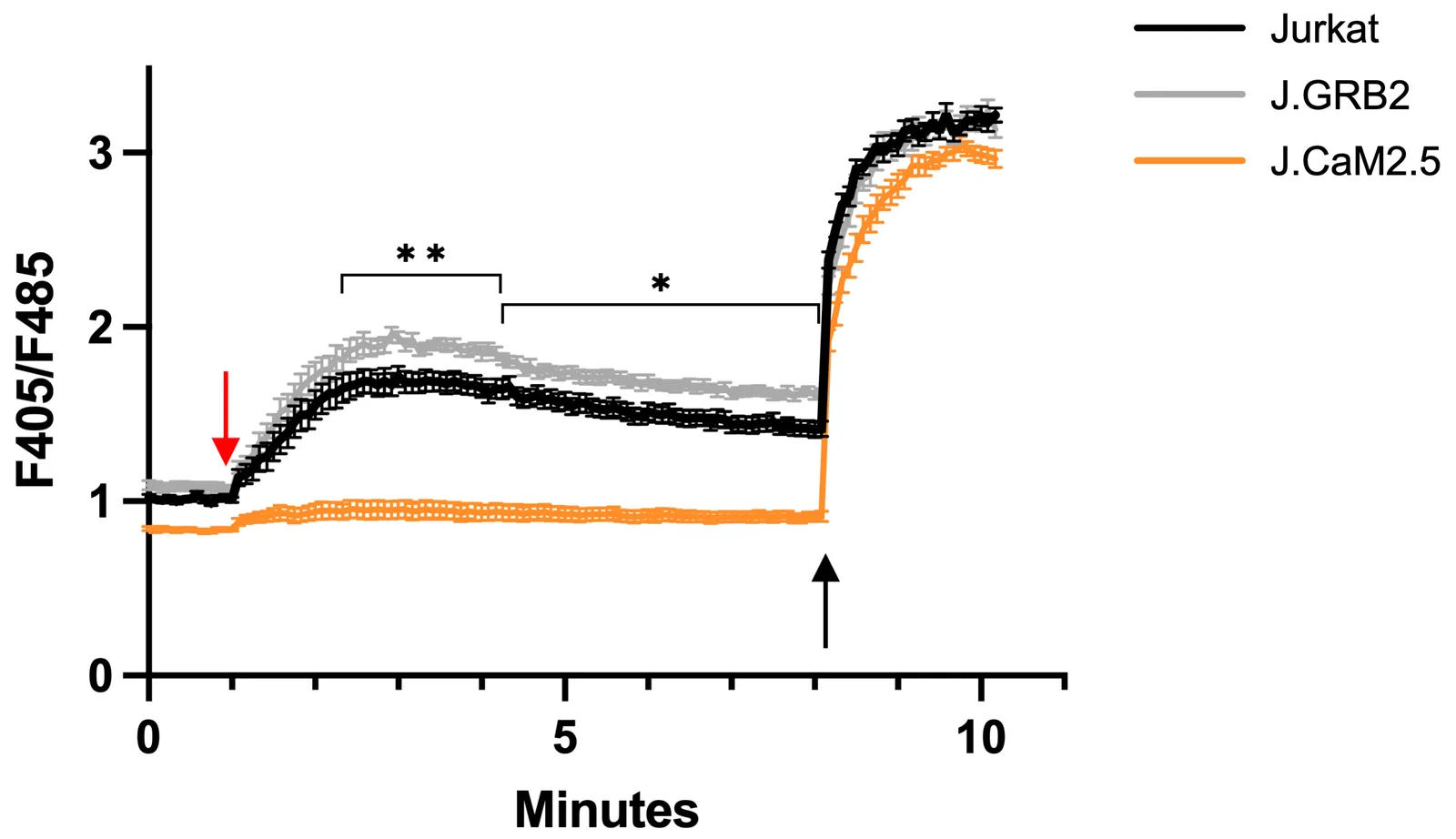
**Ca^2+^ mobilization in response to anti-CD3 stimulation in Jurkat and GRB2-deficient cells.** Intracellular calcium release (expressed as fluorescence ratio F405/F485) over time in wild-type Jurkat cells (black line), GRB2-deficient J.GRB2 cells (grey line), and J.CaM2.5 negative control cells (orange line). Anti-CD3 stimulation was applied at the time indicated by the red arrow (~1 min). Ionomycin was added near the end of the trace (~8 min) as a positive control (black arrow). Data represent the mean ± SEM from 21 experiments across 7 independent biological replicates. Statistically significant differences between Jurkat and J.GRB2 cells across the indicated time windows are denoted by asterisks (* *p* < 0.05; ** *p* < 0.01).

Having established that loss of GRB2 hyperactivates PLC-γ1, we next investigated the functional consequences of this deregulation on downstream second-messenger pathways, starting with intracellular calcium (Ca^2+^) mobilization. Parental Jurkat, GRB2-deficient (J.GRB2), and LAT-deficient (J.CaM2.5) cells were loaded with the ratiometric Ca^2+^ indicator Indo-1AM and stimulated with the anti-CD3 monoclonal antibody OKT3. As expected, LAT-deficient J.CaM2.5 cells failed to elicit any detectable increase in cytoplasmic Ca^2+^ following CD3 engagement, serving as a negative control for TCR-mediated signaling (Figure 6, orange trace). In contrast, both parental Jurkat and J.GRB2 cells displayed a rapid initial rise in cytosolic Ca^2+^ levels upon stimulation, exhibiting comparable onset kinetics during the immediate phase of the response. However, following this initial peak, GRB2-deficient cells maintained a significantly elevated plateau of cytosolic Ca^2+^ compared to parental controls (Figure 6, grey vs. black traces). Statistical analysis across 21 technical replicates from 7 independent experiments confirmed that J.GRB2 cells exhibited significantly enhanced calcium mobilization during both the peak response and the sustained plateau phase. To confirm that the maximum capacity for calcium storage and influx remained intact, cells were subsequently treated with the calcium ionophore ionomycin (~500 s), which elicited equivalent maximal fluorescence shifts across all cell lines. These data demonstrate that the heightened PLC-γ1 activation in GRB2-deficient cells translates into potentiated and sustained intracellular calcium mobilization upon TCR stimulation, paving the way to assess its downstream impact on MAPK/ERK pathway signaling.

**Figure 7:**
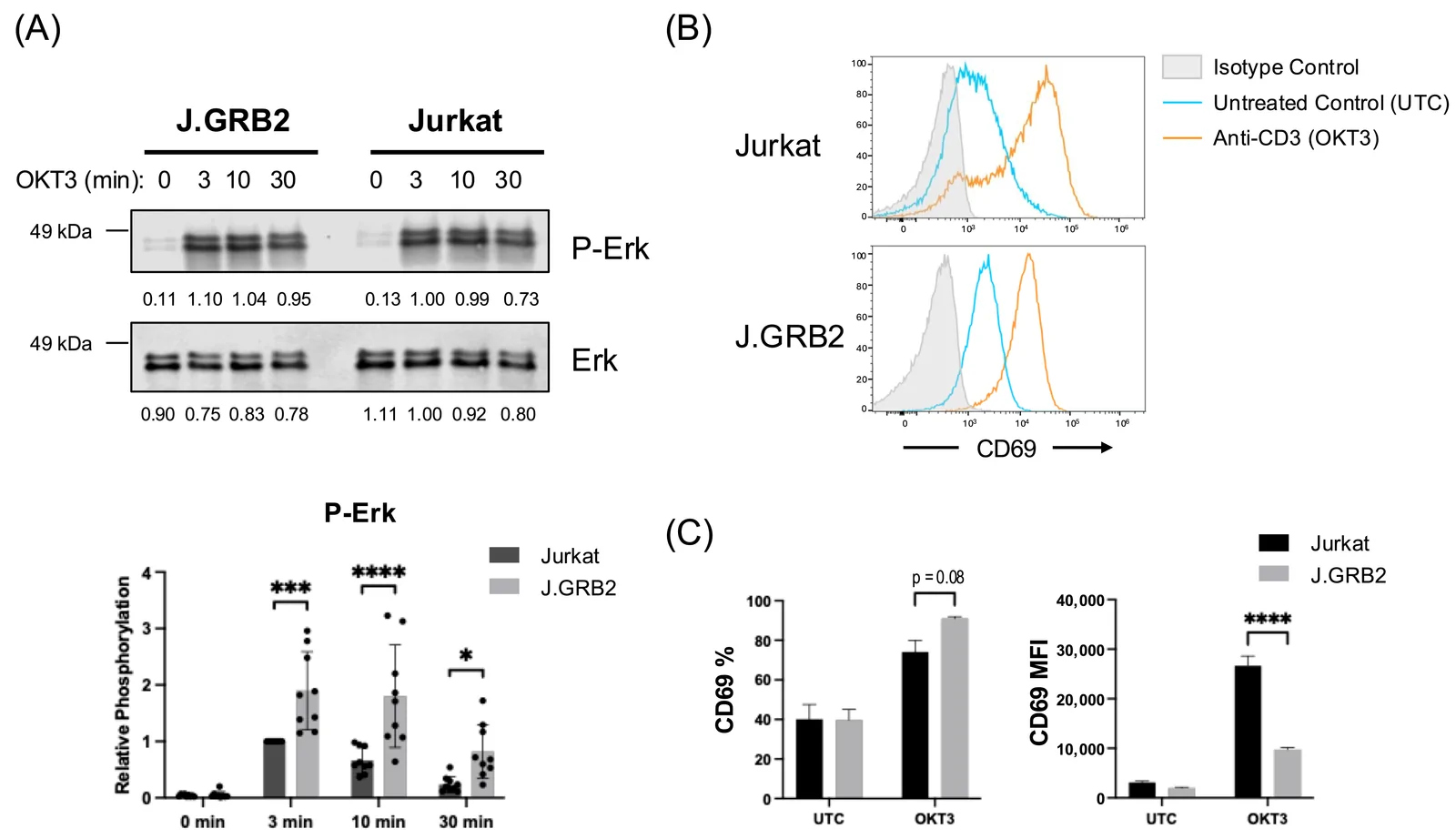
**Analysis of Erk phosphorylation and CD69 expression upon anti-CD3 stimulation in Jurkat and J.GRB2 cells.** (**A**) Representative Western blot (top) and quantitative analysis (bottom) of Erk phosphorylation (P-Erk) relative to total Erk in J.GRB2 and wild-type Jurkat cells following anti-CD3 stimulation for the indicated time points (0, 3, 10, and 30 min). Bar graphs represent the quantitative summary of 11 independent experiments. (**B**) Representative flow cytometry histograms showing surface CD69 expression in Jurkat (top) and J.GRB2 (bottom) cells. Filled grey histograms represent the isotype control, blue lines represent untreated control cells (UTC), and orange lines represent anti-CD3-treated cells. (**C**) Quantification of CD69 surface expression shown as percentage of CD69-positive cells (CD69 %, left) and mean fluorescence intensity (CD69 MFI, right) in untreated (UTC) and anti-CD3-stimulated (OKT3) conditions. Bar graphs represent the mean ± SEM from 6 independent experiments. Statistically significant differences are indicated (* *p* < 0.05; *** *p* < 0.001; **** *p* < 0.0001).

Given the sustained calcium mobilization observed in GRB2-deficient cells, we next assessed whether this hyperactivation propagates to downstream MAPK signaling by evaluating extracellular signal-regulated kinase (Erk) activation. Parental Jurkat and J.GRB2 cells were stimulated with OKT3 over a 30-min time course, and ERK phosphorylation status was analyzed by immunoblotting. While CD3 engagement induced robust ERK activation in both cell lines, GRB2 deficiency resulted in significantly augmented ERK phosphorylation (Figure 7A). Densitometric quantification across 11 independent experiments confirmed a statistically significant elevation in relative P-ERK levels in J.GRB2 cells at peak activation time points, specifically at 3 min (* *p* < 0.05) and 10 min (*** *p* < 0.001) post-stimulation (Figure 7A, bottom panel).

Finally, we examined the functional biological consequence of this enhanced signaling on T-cell activation by measuring surface expression of the early activation marker CD69 by flow cytometry. Following anti-CD3 stimulation, both parental Jurkat and J.GRB2 cells showed a marked shift in surface CD69 expression relative to untreated controls (Figure 7B). Quantification across 6 independent experiments demonstrated a slight increase in the overall percentage of CD69-positive cells in J.GRB2 mutants compared to controls, though this trend did not reach statistical significance (*p* = 0.08; Figure 7C, left panel). Strikingly, however, evaluation of mean fluorescence intensity (CD69 MFI) revealed a drastic reduction in CD69 density per cell in GRB2-deficient cells relative to parental Jurkat controls upon CD3 stimulation (**** *p* < 0.0001; Figure 7C, right panel).

Taken together, these results demonstrate that while GRB2 deficiency potentiates proximal signaling outputs, including ZAP-70, PLC-*γ*1, Ca^2+^ mobilization, and ERK phosphorylation, it paradoxically impairs the magnitude of downstream CD69 protein expression, uncoupling hyperactive proximal TCR signaling from complete transcriptional/translational activation outputs.

### 3.4. GRB2-Deficient Cells Exhibit a Concomitant Loss of THEMIS Expression

THEMIS is a key negative regulator of TCR signaling that depends on its constitutive interaction with the SH3 domain of GRB2 for recruitment to the LAT signalosome and assembly of the THEMIS-GRB2-SHP1 complex. Because THEMIS relies on GRB2 for its recruitment, we sought to investigate whether the signaling hyper-activation observed in GRB2-deficient T cells was linked to alterations in THEMIS expression. To address this, we evaluated the protein levels of THEMIS in wild-type and GRB2-knockout Jurkat T cells.

**Figure 8:**
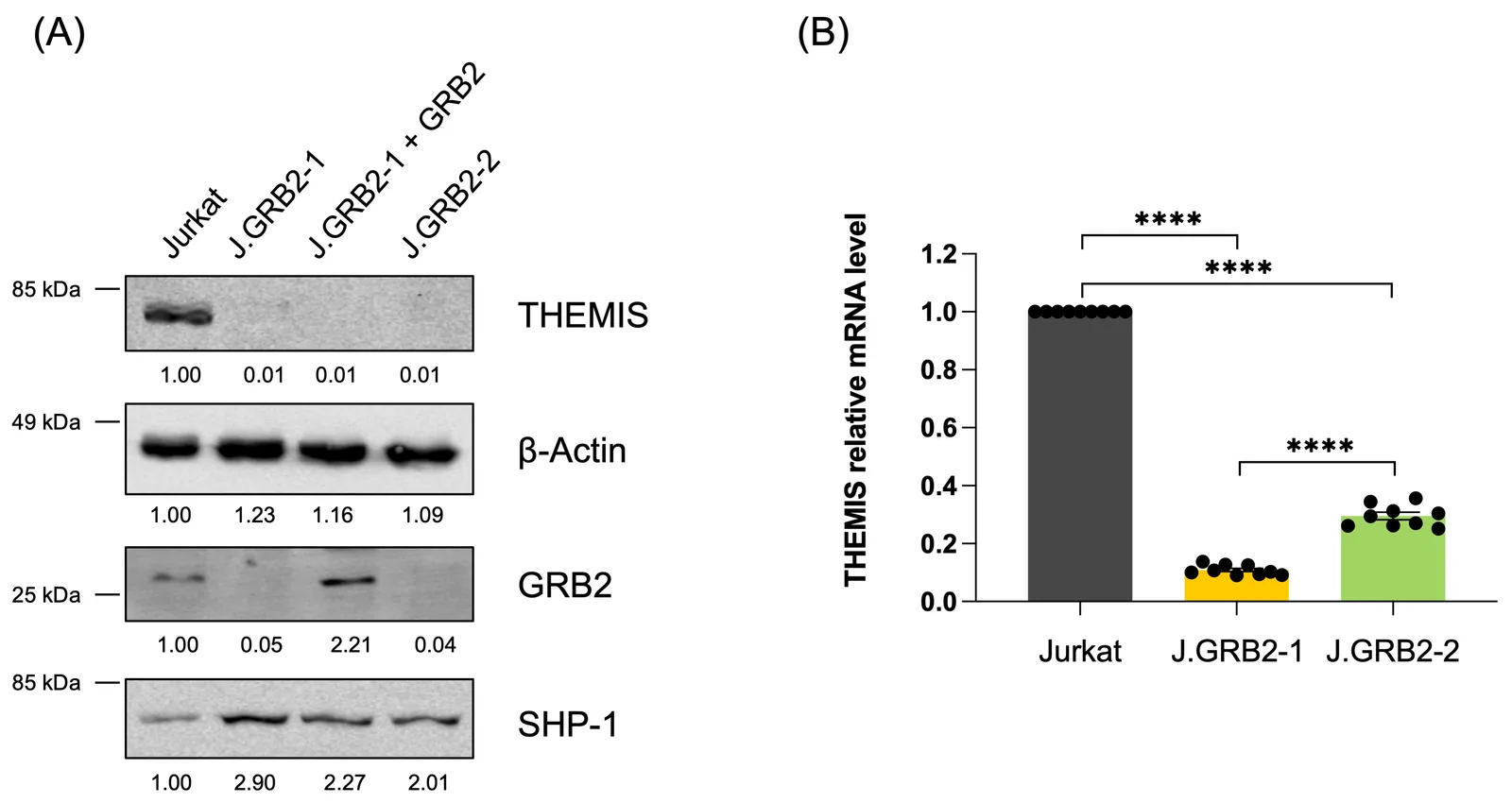
**Assessment of THEMIS expression at protein and mRNA levels in GRB2-deficient Jurkat cell lines.** (**A**) Western blot analysis showing expression of THEMIS, GRB2, and SHP-1 across parental Jurkat, GRB2-deficient clones (J.GRB2-1 and J.GRB2-2), and a reconstituted clone (J.GRB2-1 + GRB2, stably transduced via lentivirus to re-express GRB2). Whole-cell lysates (1 × 10^6^ cells/lane) were probed with the indicated antibodies, with β-actin serving as a loading control. Numerical values below the blots represent relative band intensity values for each lane. (**B**) RT-qPCR quantification of relative THEMIS mRNA levels normalized to *HPRT1* in Jurkat, J.GRB2-1, and J.GRB2-2 cells. Bars represent the mean ± standard error of the mean (SEM) from n = 9 independent experiments. **** *p* < 0.001 vs. parental Jurkat control.

Western blot analysis revealed a striking absence of THEMIS protein expression in both independent GRB2-deficient clones (J.GRB2-1 and J.GRB2-2) compared to parental Jurkat cells (Figure 8A). In contrast, the protein levels of SHP-1, another key component of the regulatory trimolecular complex, remained unchanged across all cell lines, demonstrating that this loss is specific to THEMIS rather than a global downregulation of LAT-associated signaling molecules (Figure 8A). Remarkably, stable re-expression of GRB2 via lentiviral transduction in the knockout line (J.GRB2-1 + GRB2) successfully restored GRB2 protein levels but failed to rescue THEMIS protein expression (Figure 8A). To determine whether the loss of THEMIS protein was accompanied by lower transcript levels, we measured relative THEMIS mRNA levels by RT-qPCR. Consistent with the protein data, THEMIS transcript levels were drastically decreased in both J.GRB2-1 and J.GRB2-2 clones compared to parental Jurkat controls (*p* < 0.001, Figure 8B). Taken together, these findings show that THEMIS is markedly downregulated at the mRNA level in these GRB2-knockout cell lines, although this reduction occurs independently of acute GRB2 rescue.

## 4. Discussion

Mechanistic insights into T-cell receptor signaling rely heavily on targeted loss-of-function models; here, we present the generation and functional validation of a stable GRB2-deficient Jurkat cell line developed via CRISPR/Cas9 nickase editing. We have generated two independent cell lines defective for GRB2 expression and verified the loss of the GRB2 protein in both cell lines due to insertions and deletions in both alleles that introduce premature stop codons. Until recently, there were no cell line models deficient in GRB2 expression.

Using these new CRISPR-edited lines, we systematically mapped the functional consequences of complete GRB2 loss on TCR-proximal activation dynamics. Functional analysis revealed that GRB2 knockout leads to marked hyperactivation of ZAP-70 at its activating Tyr319 residue, reinforcing the role of GRB2 as a key negative regulator of upstream kinase activity. However, downstream examination of the LAT signalosome uncovered a global reduction in LAT phosphorylation: phosphorylation across all major tyrosine residues (Tyr132, Tyr171, and Tyr191) was significantly impaired upon GRB2 deficiency. Strikingly, despite this widespread suppression of LAT phosphorylation, loss of GRB2 failed to dampen downstream signaling and instead triggered robust hyperphosphorylation of PLC-γ1 and heightened intracellular Ca^2+^ influx upon TCR engagement.

Our findings build upon prior work showing that knocking down GRB2 in primary human CD4^+^ T cells and HuT78 T-cell lines enhances early signaling events (such as the phosphorylation of Lck, ζ-chain, ZAP-70, and SLP-76), while simultaneously impairing downstream outputs like PLC-γ1 phosphorylation, intracellular calcium mobilization, and IL-2/IFN-γ release [14]. While this dual action underscored GRB2’s involvement in T-cell fine-tuning, the precise molecular basis for these uncoupled effects has yet to be fully elucidated. The general reduction in LAT phosphorylation (at Tyr132, Tyr171, and Tyr191) observed in our GRB2-deficient cells provides a potential molecular mechanism for the signaling reorganization described by Ruminski et al. [23]. Using quantitative mass spectrometry, they showed that loss of GRB2 triggers extensive molecular remodeling of the SLP-76 signaling network. Because these LAT tyrosine residues (particularly Tyr171, Tyr191, and Tyr226) serve as primary docking sites for GRB2-family adaptors and their associated negative regulators, a widespread decrease in LAT phosphorylation could disrupt the recruitment of negative feedback complexes. This structural altered state within the LAT/SLP-76 interactome likely explains why PLC-γ1 activation is uncoupled from overall LAT phosphorylation status and hyperactivated in the absence of GRB2.

The observed decrease across all major LAT phosphorylation sites in GRB2-deficient cells raises intriguing questions regarding local receptor architecture, kinase accessibility, and phosphatase dynamics. One plausible explanation is that GRB2 binding natively acts as a steric shield, protecting phosphorylated tyrosine residues from rapid dephosphorylation by protein tyrosine phosphatases (PTPs). In the complete absence of GRB2, these exposed phosphotyrosines may become significantly more accessible to cytosolic phosphatases, leading to accelerated dephosphorylation kinetics following TCR engagement. Alternatively, GRB2 loss could alter the spatial clustering or stability of LAT within lipid rafts, reducing its accessibility to active ZAP-70.

Unexpectedly, PLC-γ1 exhibited enhanced phosphorylation and downstream signaling despite a reduction in LAT Tyr132 phosphorylation, its canonical docking site. This paradox, however, highlights the dual role of GRB2 as both a physical scaffold for activation networks and a critical recruiter of negative feedback loops. Because GRB2 constitutively bridges LAT phosphotyrosine residues (particularly Tyr171 and Tyr191) to negative regulators (such as the ubiquitin ligase Cbl and, via its SH3 domains, the THEMIS–SHP1 complex), the complete loss of GRB2 abolishes recruitment of these negative-feedback effectors to the LAT signalosome regardless of residual LAT phosphorylation. While the concomitant reduction in LAT phosphorylation observed across all sites (Y132, Y171, and Y191) may further influence this axis, the principal driver of impaired negative-feedback recruitment is the absence of the GRB2 scaffold itself. Unchained from GRB2-mediated negative feedback, upstream kinases like Lck remain hyperactive, driving sustained ZAP-70 Tyr319 phosphorylation. At the same time, the loss of tonic inhibitory restraints (such as GRB2-mediated competitive SOS1 binding or GADS/SLP-76 negative feedback tuning) permits hyper-efficient PLC-γ1 docking and activation, even when Tyr132 phosphorylation is significantly blunted and overall LAT phosphorylation is reduced. Thus, the loss of GRB2 disrupts the self-limiting brake of the TCR signaling cascade without impairing the core signalosome, triggering hyperactivated downstream calcium and MAPK pathways despite a global reduction in LAT phosphorylation.

Finally, our phenotypic evaluation of both GRB2-deficient Jurkat lines revealed important insights regarding the integrity of the SHP-1 inhibitory axis (Figure 8). Immunoblot analysis demonstrated that SHP-1 protein expression remains fully intact and comparable to parental Jurkat cells across both GRB2-deficient clones (J.GRB2-1 and J.GRB2-2), confirming that loss of GRB2 does not downregulate SHP-1 itself (Figure 8A). In contrast, both GRB2-deficient lines exhibited a striking, near-complete loss of THEMIS protein expression (Figure 8A), accompanied by a significant reduction in *THEMIS* relative mRNA levels (Figure 8B). To test whether GRB2 is strictly required to stabilize THEMIS protein or drive its expression, we performed lentiviral rescue by reconstituting GRB2 expression in the J.GRB2-1 line (J.GRB2-1 + GRB2). Re-expression of GRB2 failed to restore THEMIS protein levels (Figure 8A), indicating that the loss of THEMIS expression is not an acute, reversible protein instability phenotype caused by the physical absence of GRB2, but instead reflects transcriptional suppression or a clonal feature acquired during gene editing. Because THEMIS recruitment to LAT is strictly dependent on its constitutive interaction with GRB2, and GRB2 is itself required to bridge SHP-1 to THEMIS and deliver the complex to the LAT signalosome, the concomitant loss of both GRB2 and THEMIS is expected to abolish the local, signalosome-associated pool of SHP-1 activity, independently of total cellular SHP-1 abundance. This provides a mechanism-based explanation for the hyperactivation of the ZAP-70, PLC-γ1, and ERK signaling pathways observed in GRB2-deficient cells, complementing the other negative-feedback mechanisms discussed above (SOS1 competition, GADS/SLP-76 tuning, Cbl-mediated ubiquitination).

In summary, the establishment of these GRB2-deficient Jurkat cell lines provides a robust, clean knock-out platform that resolves long-standing questions regarding the dual adapter and regulatory roles of GRB2 in T cells. By demonstrating that complete GRB2 loss leads to attenuated phosphorylation across the LAT signalosome, while simultaneously amplifying ZAP-70, PLC-γ1, and downstream pathway activation, our study highlights GRB2 as an essential mediator of negative feedback integration at this signaling hub. These findings refine current models of TCR signal regulation, establishing that GRB2 is dispensable for core LAT signalosome assembly but vital for maintaining the self-limiting feedback threshold that prevents aberrant T-cell hyperactivation.

## 5. Conclusions

The generation and functional validation of stable, CRISPR/Cas9-edited GRB2-deficient Jurkat cell lines establish a somatic knockout platform to dissect the complex, multi-tiered functions of GRB2 in human T cells. By achieving complete loss of GRB2, our study resolves key ambiguities surrounding its dual role as both a physical scaffold and an essential coordinator of negative feedback. We demonstrate that while GRB2 deficiency leads to attenuated phosphorylation across the LAT signalosome (likely due to increased exposure to cytosolic phosphatases or loss of steric shielding), it simultaneously triggers marked hyperactivation of upstream ZAP-70 and downstream PLC-*γ*1, Erk, and calcium signaling cascades. This profound uncoupling reveals that GRB2 is functionally dispensable for downstream cascade execution, but acts as a critical, non-redundant negative regulator required to restrain proximal TCR activation kinetics.

Furthermore, our characterization of SHP-1 and THEMIS dynamics provides vital mechanistic context regarding how this negative feedback regulation is compromised. Although SHP-1 phosphatase remains stably expressed, its inability to suppress signaling in GRB2-deficient cells underscores that SHP-1 function relies strictly on GRB2 to act as a physical bridge for its recruitment to LAT. Without the GRB2 scaffold and compounded by the concurrent loss of THEMIS protein and mRNA expression, SHP-1 is functionally decoupled from the LAT signalosome and unable to dephosphorylate its proximal substrates. Consequently, our results demonstrate that GRB2 exerts direct, indispensable negative regulatory control by anchoring inhibitory feedback complexes to the TCR signaling apparatus. Taken together, these findings refine our understanding of T-cell threshold tuning and highlight GRB2-mediated feedback loops as potential targets for modulating T-cell sensitivity in immunotherapeutic applications.

## Data Availability

The original contributions presented in this study are included in the article. Further inquiries can be directed to the corresponding author.

## Supplementary Materials

The following supporting information can be downloaded at https://www.mdpi.com/article/10.3390/biology15212027/s1.

## Abbreviations

The following abbreviations are used in this manuscript:

ANOVA	Analysis of Variance
CRISPR	Clustered Regularly Interspaced Short Palindromic Repeats
Gads	GRB2-related Adaptor Downstream of Shc
GRB2	Growth Factor Receptor-Bound Protein 2
LAT	Linker for Activation of T Cells
PLC-γ1	Phospholipase C Gamma 1
SLP-76	SH2 Domain-Containing Leukocyte Protein of 76 kDa
T7E1	T7 Endonuclease I
TCR	T-Cell Receptor
THEMIS	Thymocyte-Expressed Molecule Involved in Selection
ZAP-70	Zeta-Chain-Associated Protein Kinase 70
